# Discovery of the rare genus *Blacometeorus* Tobias, 1976 (Hymenoptera, Braconidae, Blacinae) in the Oriental part of China, with description of a new species

**DOI:** 10.3897/zookeys.65.451

**Published:** 2010-10-29

**Authors:** Hong-fei Chai, Jun-hua He, Xue-xin Chen

**Affiliations:** State Key Lab of Rice Biology, Institute of Insect Sciences, Zhejiang University, 268 Kaixuan Road, Hangzhou 310029, China

**Keywords:** Hymenoptera, Braconidae, Blacinae, *Blacometeorus sinicus*, Oriental, new species

## Abstract

The rare genus Blacometeorus Tobias, 1976 (Hymenoptera: Braconidae: Blacinae)is discovered in Yunnan, China, and a new species, Blacometeorus sinicus Chai & Chen, **sp. n.** is described and illustrated. It represents the first record of this genus both for China and Oriental region. A key to genus Blacometeorus is updated.

## Introduction

The Blacinae Foerster is a cosmopolitan subfamily of Braconidae (Hymenoptera) with five tribes, 14 genera and 207 species known in the world (Yu et al., 2005). Blacometeorus Tobias, 1976 is the only genus of the Blacini with vein r-m of fore wing present (Tobias, 1976; van Achterberg, 1988). Four species of Blacometeorus are reported to date, i.e., Blacometeorus brevicauda (Hellén, 1958), Blacometeorus intermedius Tobias, 1976, Blacometeorus pusillus (Hellén, 1958) and Blacometeorus konishii Belokobylskij, 2000, all from the Palaearctic region, but they are rarely collected. Recently a specimen of Blacometeorus was discovered in Yunnan, China, representing a new species, Blacometeorus sinicus Chai & Chensp. n., which we describe and illustrate. It represents the first record of this genus both for China and Oriental region.

## Materials and methods

The terminology and measurements used follow van Achterberg (1976, 1988). Additional sources for the description of ocelli and tentorial pits are Belokobylskij (2000). The descriptions and measurements were made under the Leica MZ 12.5 and Zeiss Stereo Discovery V8 microscope, and photos taken by a digital camera (Q-Imaging, Micropublisher 3.3 RTV) attached to a Leica MZ APO stereomicroscope (Wetzlar, Germany) using Synoptics Auto-Montage version 5.0 software. Type specimen is deposited in the Parasitic Hymenoptera Collection of the Zhejiang University, Hangzhou, China (ZJUH).

## Taxonomy

### Blacometeorus sinicus

Chai & Chen sp. n.

urn:lsid:zoobank.org:act:62254724-3720-49AF-854C-7EEDAC227C6C

Figs 1–10 

#### Female.

Length of body 3.0 mm, of fore wing 2.8 mm.

**Figures 1–10.**
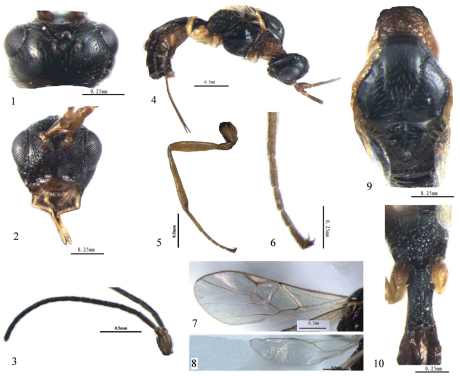
Blacometeorus sinicus Chai & Chen, sp. n. ♀, holotype. **1** antenna **2** head, frontal view **3** head, dorsal view **4** mesonotum, dorsal aspect **5** propodeum and metasomal tergite I–II, dorsal aspect **6** fore wing **7** hind wing **8** body, lateral view **9** hind leg **10**, hind tarsus.

*Head* (Figs 1–3). Head distinctly and roundly narrowed below eyes; frons and occiput weakly concave; face largely rugose; length of maxillary palp subequal to height of head; POL equal to Od, 0.5 times OOL; Ocelli almost in equilateral triangle; length of eye in dorsal view equal to temple; malar space 1.2 times as long as basal width of mandible; tentorial pits large, distance between pits 1.3 times distance from pit to eye; width of clypeus 2.5 times its median height.

Antenna slender, 17-segmented; scapus twice as long as pedicellus; third segment 1.2 times as long as fourth segment; third, fourth and penultimate segments 4.7, 4.2, and 1.7 times as long as their width, respectively; penultimate segment 0.7 times as long as the apical segment; sixth and seventh segments normal.

*Mesosoma* (Figs 4, 9, 10). Length of mesosoma 1.5 times its height; pronotum largely with rugae; scutellar sulcus deep, rugose, with a distinct medio-longitudinal carina, almost 0.7 times as long as scutellum; scutellum with fine lateral carinae, not protruding dorsally; surface of propodeum largely reticulate-rugose, anteriorly narrowly smooth, its medial area absent.

*Wings* (Figs 7, 8). Fore wing: Length of fore wing 3 times its width; pterostigma narrow, its length 5 times maximum width; first discal cell narrowly truncated; 1-CU1 : 2-CU1 = 6:21. Hind wing: 1r-m 0.9 times 1-M.

*Legs* (Figs 5, 6). Hind coxa with distinct curved dorsal keel; length of femur, tibia, and basitarsus of hind leg 4.6, 8.8 and 9.0 times their width, respectively; hind tarsus 1.1 times as long as hind tibia, its second segment 0.5 times as long as first segment.

*Metasoma* (Figs 4, 10). First tergite long, parallel-sided, with large spiracular tubercles in basal 0.35; first tergite 2.6 times as long as its apical width, largely coarsely reticulate, dorsal carinae distinct in basal half; second tergite weakly sculptured basally; ovipositor sheath 1.7 times as long as first tergite, 0.33 times as long as fore wing.

*Colour*. Dark brown; palpi and legs brownish yellow, but hind coxa, apex of hind tibia and tarsus darkened; tegulae and hypopygium brown; wing membrane subhyaline; pterostigma light brown; veins brown.

#### Male.

Unknown.

#### Type material.

 Holotype, ♀ (ZJUH): China, Yunnan, Baoshan, Lujiangba, Gaoligong Mountain Natural Park, 24°49′44″N, 98°46′04″E, 2181m elev., 11.v.2009, coll. Wang Man-man, no.200904565.

#### Diagnosis.

 The new species is similar to Blacometeorus konishii Belokobylskij, 2000, but can be separated from the latter by having the parallel-sided first metasomal tergite with big spiracular tubercles in basal 0.35, the second tergite weakly sculptured basally, the length of ovipositor sheath 0.33 times as long as fore wing, the length of scapus 2 times length of pedicellus, and the vein r-m of fore wing pigmented.

#### Biological notes.

Nothing is known about the host of this species.

#### Etymology.

From Latin “sinicus” (Chinese), referring to the first discovery in China.

## Key to species of genus Blacometeorus Tobias, 1976

**Table:** 

1.	First metasomal tergite distinctly widened apically, its length about 1.5 times (+) its apical width and extensively sculptured; third antennal segment 3–3.3 (+) times as long as its width, sixth and seventh segments of + very short; vein 1r-m of hind wing 1.3–1.6 times as long as vein 1-M; ovipositor sheath 0.25–0.29 times as long as fore wing; hind tarsus infuscated; Palaearctic: United Kingdom, Czechoslovakia, Finland, Germany, and Russia (including Primorskiy kray)	Blacometeorus brevicauda (Hellen)
–	First tergite (sub) parallel-sided, its length 1.6–3 times its apical width; if about 1.6 times then only medially sculptured and hind tarsus yellowish; length of third antennal segment 3.8 (>)-4.7 (+) times its width, sixth and seventh segments of + less shortened; length of vein 1r-m of hind wing 0.9–1.2 times vein 1-M; length of ovipositor sheath 0.35–0.45 times fore wing (unknown in *intermedius*)	2
2.	Scapus (without radix) somewhat longer (about 1.2 times) than pedicellus; face completely smooth; first metasomal tergite 1.6–1.9 times (>) as long as its apical width; scutellum without lateral carinae, somewhat protruding dorsally; Palaearctic: Azerbaijan, Czechoslovakia	Blacometeorus intermedius Tobias
–	Scapus much longer than pedicellus; face with rugae or striation; first tergite 2.2–2.8 times (+) its apical width; scutellum with fine lateral carinae, not protruding dorsally	3
3	Face largely smooth, except some rugae near toruli; distance between hind ocelli much longer than diameter of posterior ocellus; first metasomal tergite short, its length 2.2 times its apical width; Palaearctic: United Kingdom, Finland, Hungary	Blacometeorus pusillus (Hellen)
–	Face punctulate and rugose, with striation partly; distance between hind ocelli equal to diameter of posterior ocellus; first metasomal tergite long, its length 2.6–2.8 times its apical width	Blacometeorus pusillus (Hellen)
–	Face punctulate and rugose, with striation partly; distance between hind ocelli equal to diameter of posterior ocellus; first metasomal tergite long, its length 2.6–2.8 times its apical width	4
4	First metasomal tergite weakly narrowed apically, with small spiracular tubercles in basal 0.35; second tergite smooth; ovipositor sheath 0.42 times as long as fore wing; scapus 4 times as long as pedicellus; vein r-m of fore wing unpigmented; Palaearctic: Japan	Blacometeorus konishii Belokobylskij
–	First metasomal tergite parallel-sided, with large spiracular tubercles in basal 0.35; second tergite weakly sculptured basally; length of ovipositor sheath 0.33 times as long as fore wing; length of scapus 2 times length of pedicellus; vein r-m of fore wing pigmented; Oriental: China	Blacometeorus sinicus sp. n.

## Supplementary Material

XML Treatment for Blacometeorus sinicus
